# Propensity score analysis of non‐anatomical *versus* anatomical resection of colorectal liver metastases

**DOI:** 10.1002/bjs5.50154

**Published:** 2019-03-18

**Authors:** K. M. Brown, M. F. Albania, J. S. Samra, P. J. Kelly, T. J. Hugh

**Affiliations:** ^1^ Upper Gastrointestinal Surgical Unit, Royal North Shore Hospital and North Shore Private Hospital, St Leonards Sydney New South Wales Australia; ^2^ Sydney School of Public Health, University of Sydney Sydney New South Wales Australia; ^3^ Discipline of Surgery, Northern Clinical School, University of Sydney Sydney New South Wales Australia; ^4^ Faculty of Medicine and Health Sciences Macquarie University Sydney New South Wales Australia

## Abstract

**Background:**

There are concerns that non‐anatomical resection (NAR) worsens perioperative and oncological outcomes compared with those following anatomical resection (AR) for colorectal liver metastases (CRLM). Most previous studies have been biased by the effect of tumour size. The aim of this study was to compare oncological outcomes after NAR *versus* AR.

**Methods:**

This was a retrospective study of consecutive patients who underwent CRLM resection with curative intent from 1999 to 2016. Data were retrieved from a prospectively developed database. Survival and perioperative outcomes for NAR and AR were compared using propensity score analyses.

**Results:**

Some 358 patients were included in the study. Median follow‐up was 34 (i.q.r. 16–68) months. NAR was associated with significantly less morbidity compared with AR (31·1 *versus* 44·4 per cent respectively; *P* = 0·037). Larger (hazard ratio (HR) for lesions 5 cm or greater 1·81, 95 per cent c.i. 1·13 to 2·90; *P* = 0·035) or multiple (HR 1·48, 1·03 to 2·12; *P* = 0·035) metastases were associated with poor overall survival (OS). Synchronous (HR 1·33, 1·01 to 1·77; *P* = 0·045) and multiple (HR 1·51, 1·14 to 2·00; *P* = 0·004) liver metastases, major complications after liver resection (HR 1·49, 1·05 to 2·11; *P* = 0·026) or complications after resection of the primary colorectal tumour (HR 1·51, 1·01 to 2·26; *P* = 0·045) were associated with poor disease‐free survival (DFS). AR was prognostic for poor OS only in tumours smaller than 30 mm, and R1 margin status was not prognostic for either OS or DFS. NAR was associated with a higher rate of salvage resection than AR following intrahepatic recurrence.

**Conclusions:**

NAR has at least equivalent oncological outcomes to AR while proving to be safer. NAR should therefore be the primary surgical approach to CRLM, especially for lesions smaller than 30 mm.

## Introduction

The conceptual aim of parenchymal‐sparing or non‐anatomical resection (NAR) for colorectal liver metastases (CRLM) is to preserve as much functional parenchyma as possible in order to decrease the risk of postoperative liver failure and facilitate future repeat resection in the event of intrahepatic recurrence^1^, ^2^, ^3^. Quantifying the effect of the surgical approach on morbidity and mortality, as well as disease‐free survival (DFS) and overall survival (OS) has been difficult. Data from studies published to date have been conflicting, with some favouring NAR^2^, ^4^, ^5^ whereas others support anatomical resection (AR)^6^, ^7^.

A central issue in the debate is the possible confounder that large tumours may inherently be more prone to compromising vital inflow or outflow structures than small tumours, thereby necessitating AR. Differences in tumour size between groups may also reflect different tumour biology. Numerous previous studies^8^, ^9^, ^10^, ^11^ comparing AR and NAR did not account for tumour size and number, or failed adequately to control for the potential effect of these variables in multivariable analysis. Several groups have attempted to circumnavigate this by either limiting the analysis to small solitary (less than 30 mm)^2^ or multiple^12^ tumours, or performing case‐controlled analyses^5^, ^13^. This may, however, result in selection bias for good prognostic lesions, and so conclusions may not be applicable to the full spectrum of disease. Other studies have failed to define AR or had substantial missing data. Consequently, there is ongoing controversy about whether NAR for CRLM leaves undetected residual disease, compromises resection margins, or increases the rate of intrahepatic recurrence^14^, ^15^.

The hypothesis of the present study was that resection type (anatomical or non‐anatomical) does not influence oncological outcomes after liver resection for CRLM as long as an R0 resection margin is achieved. The aim of this study was to investigate the impact of resection type on short‐ and long‐term outcomes by controlling for tumour size and number after applying a propensity score (PS) analysis to the final model using inverse probability of treatment weighting (IPTW).

## Methods

This was a retrospective study with data from a prospectively developed database. Included were consecutive patients with CRLM who underwent liver resection with curative intent from January 1999 to December 2016 at the Northern Upper Gastrointestinal Surgery Unit, Royal North Shore and North Shore Private Hospitals, Sydney, New South Wales, Australia. This expands on previous work^16^ published by the authors' group, by including a larger number of patients as well as the corresponding primary tumour variables. Patients were excluded if they had simultaneous AR and NAR, or an R2 resection. Those who underwent repeat liver resections were included in the analyses but were censored on the date of detection of their recurrence. Ethical approval for the study was provided by the Human Research Ethics Committee of the Northern Sydney Local Health District and the North Shore Private Hospital Ethics Committee.

### Resection technique and perioperative factors

Liver resection nomenclature was documented as per the Brisbane 2000 terminology^17^. NAR was defined as resection of a lesion without regard to hepatic segmental anatomy. AR was defined by specific procedure types including: extended right or left hepatectomy, right or left hepatectomy, central liver resection, right posterior sectionectomy, right anterior sectionectomy, left lateral sectionectomy or caudate resection. ARs were further assigned an externally validated complexity score^18^. Primary colorectal cancer factors included Dukes' class and complications (if any) after the primary colorectal resection. Preoperative chemotherapy was with 5‐fluorouracil and platinum‐based regimens according to local protocols.

All procedures were either performed or supervised by two experienced hepatobiliary surgeons. The choice of AR *versus* NAR was determined by the operating surgeon using a combination of preoperative and intraoperative evaluation. Patients with limited extrahepatic, intra‐abdominal disease (such as porta‐hepatis lymph node involvement or isolated upper‐quadrant peritoneal disease) were not excluded from resection. Liver transection was performed using the Cavitron Ultrasonic Aspirator® (CUSA) dissection device (Integra Life Sciences, Plainsboro, New Jersey, USA) under low central venous pressure with intermittent inflow occlusion.

R1 resection was defined as a microscopically positive margin. Perioperative morbidity was recorded. The highest grade complication was recorded for each patient using the Clavien–Dindo classification^19^. Major complications were those requiring surgical, endoscopic or radiological intervention (grade III) and ICU management (grade IV). Perioperative mortality referred to death during the same admission (in‐hospital) or within 90 days of surgery. Liver‐related complications were documented according to International Study Group of Liver Surgery definitions^20^, ^21^.

### Patient follow‐up

After initial postoperative review at 1 month, all patients were followed up at 6‐month intervals, and annually indefinitely after 5 years. At each visit serum was obtained for liver function tests and estimation of tumour markers. In addition, CT of the abdomen and thorax was performed annually. Triple‐phase contrast‐enhanced MRI and PET were performed to define lesions considered suspicious on CT or with rising serum tumour markers.

### Statistical analysis

OS was defined as the time from hepatic resection to date of death (all‐cause mortality). DFS was defined as the time from liver surgery to date of either death or first evidence of recurrence (intrahepatic or extrahepatic). Patients who died during surgery were excluded from survival analyses.

Demographic descriptive statistics were reported using mean(s.d.) and median (i.q.r.) values depending on the distribution. Categorical variables were compared with the χ^2^ test. Continuous variables were compared using a two‐sided Student's *t* test or Mann–Whitney *U* test as appropriate. Kaplan–Meier curves were constructed for OS and DFS, and univariable survival analysis was performed using the log rank test.

To account for missing data, multiple imputation was used^22^. Twenty imputed data sets were created using the chained equations method. All prediction equations included age at operation, sex, temporal relationship, size of largest tumour, number of tumours, liver resection complications, resection margin, the censor variable for OS, the censor variable for DFS, the log of OS and the log of DFS time. Given the number of missing observations for tumour differentiation and serum carcinoembryonic antigen levels, these were not included in subsequent analyses. To assess the validity of the imputed data, the distributions of imputed, completed and observed data were compared. In addition, trace plots of means and standard deviations of the imputed values were generated to assess convergence of the 20 imputed chain equations.

To control for confounding imbalances in baseline co‐variables between the AR and NAR groups, an IPTW method of PS analysis was used^23^, ^24^. Co‐variables included in the PS model were age, sex, year of liver operation, synchronicity, whether the patient received preoperative chemotherapy, number of tumours, maximum tumour size and use of the Pringle manoeuvre. The PS for each patient was then averaged across the imputed data sets^25^ before being used to calculate a stabilized IPTW. IPTW‐adjusted Kaplan–Meier curves were then created^26^, and difference in survival was tested using a Cox proportional hazards (CPH) model. ‘Doubly robust’ multivariable CPH models^27^ were then built by applying the IPTW and adjusting for potentially significant co‐variables identified in the univariable analysis (*P* < 0·250), including tumour size, number and margin status. The purposeful selection of co‐variables method was used to select variables for the final model, which were then assessed for the validity of the proportional hazards assumption using Shoenfeld residuals and goodness‐of‐fit using Cox–Snell residuals. For sensitivity analyses, a complete case analysis was performed using the same CPH model.

A subgroup analysis was performed according to the size of the largest CRLM (less than 30 mm *versus* 30 mm or more); the above univariable and multivariable analysis was applied separately to each of these subgroups. A threshold of 30 mm was selected based on previous literature^2^. For the subgroup PS, the co‐variables used in the treatment model were the same as those for the whole cohort, with the exception of tumour size and use of the Pringle manoeuvre.

Data management and statistical analyses were performed using Stata® SE for Windows® version 15.1 (StataCorp, College Station, Texas, USA). The statistical significance level was set at α = 0·05.

## Results

A total of 391 patients underwent liver resection during the study period. Of these, 33 were excluded because they underwent simultaneous AR and NAR (25 patients), had a squamous cell primary tumour (4) or an R2 liver resection margin (4). The remaining 358 eligible patients were included in the study; 194 (54·2 per cent) underwent AR and 164 (45·8 per cent) had NAR.

**Figure 1:**
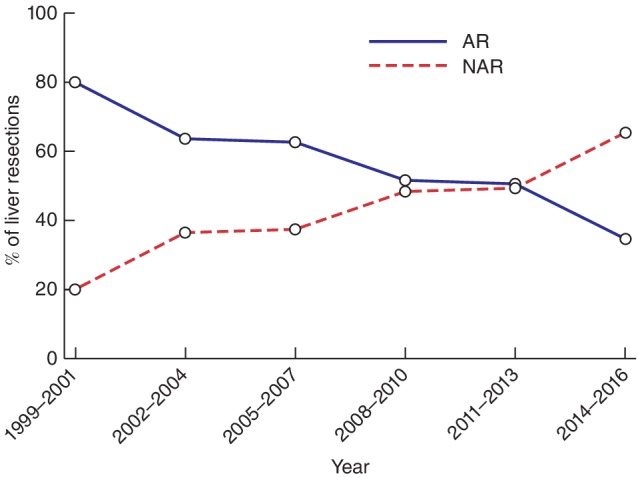
Changes in hepatic resection type over time. AR, anatomical resection; NAR, non‐anatomical resection. *P* = 0·006 (χ^2^ test)

Baseline characteristics are summarized in *Table* 1. All resected lesions were metastatic adenocarcinomas. Median follow‐up was 34 (i.q.r. 16–68) months. There was a significant increase in the proportion of NAR performed over time (*P* = 0·005) (*Fig*. 1). Some 48·8 per cent of resections in the NAR group were for solitary lesions. The proportions of types of AR are shown in *Table* S1 (supporting information). Some 139 of the 194 ARs (71·6 per cent) were of intermediate complexity, with a median complexity score of 4·87 (i.q.r. 4·39–6·21). There was no significant change in complexity score over time. The overall perioperative morbidity rate was 39·1 per cent (140 of 358), with a 9·1 per cent (57 of 353) major complication rate. There were five postoperative deaths (1·4 per cent) during the study period, all in the AR group. These deaths occurred during 2005–2007, and there were no further events in the following 9 years.

**Table 1:** Characteristics of patients in anatomical and non‐anatomical groups

	Non‐anatomical (*n* = 164)	Anatomical (*n* = 194)	*P* †
Age (years)*	64 (57–71)	66 (57–73)	0·437‡
Sex ratio (M : F)	102 : 62	123 : 71	0·814
Site of primary tumour			0·298
Colon	115 (70·1)	126 (64·9)	
Rectum	49 (29·9)	68 (35·1)	
Temporal relationship			0·577
Synchronous	86 (52·4)	96 (49·5)	
Metachronous	78 (47·6)	98 (50·5)	
Dukes' class			0·443
A/B	55 (33·5)	59 (30·4)	
C	96 (58·5)	123 (63·4)	
Missing	13 (7·9)	12 (6·2)	
Primary surgery complication (*n* = 336)	17 of 154 (11·0)	18 of 182 (9·9)	0·731
Preoperative chemotherapy	117 of 163 (71·8)	142 (73·2)	0·765
Perioperative RFA	5 (3·0)	11 (5·7)	0·241
Year of liver surgery			0·006
1999–2004	19 (11·6)	40 (20·6)	
2005–2010	77 (47·0)	101 (52·1)	
2011–2016	68 (41·5)	53 (27·3)	
Pringle manoeuvre (*n* = 324)	136 of 150 (90·7)	140 of 174 (80·5)	0·010
Tumour size (cm)*	2·5 (1·8–3·5)	4 (2·2–6·0)	< 0·001§
No. of tumours			0·724
Solitary	80 (48·8)	91 (46·9)	
Multiple	84 (51·2)	103 (53·1)	
2–3	53 (32·3)	72 (37·1)	
> 3	31 (18·9)	31 (16·0)	
Duration of surgery (min)* (*n* = 325)	150 (100–180)	210 (150–270)	< 0·001§
Estimated blood loss (ml)* (*n* = 344)	100 (50–200)	300 (120–500)	< 0·001§
Blood transfusion (*n* = 354)	1 of 163 (0·6)	21 of 191 (11·0)	< 0·001
Hospital stay (days)* (*n* = 355)	7 (6‐9)	8 (7–11)	0·003§
Complications			0·006
None or minor	147 (89·6)	149 of 189 (78·8)	
Major (excluding death)	17 (10·4)	40 (20·6)	
Postoperative bleed	0 (0)	2 (1·0)	0·190
Bile leak	4 (2·4)	13 (6·7)	0·059
Postoperative liver failure	0 (0)	5 (2·6)	0·038
Perioperative mortality	0 (0)	5 (2·6)	0·038
Resection margin			0·059
R0	144 (87·8)	156 (80·4)	
R1	20 (12·2)	38 (19·6)	
Recurrence pattern	*n* = 90	*n* = 132	0·246
Intrahepatic only	37 (41)	41 (31·1)	
Extrahepatic only	22 (24)	43 (32·6)	
Mixed intrahepatic and extrahepatic	31 (34)	48 (36·4)	
Length of follow‐up (months)*	35 (16–68)	32 (15–68)	0·51§

Values in parentheses are percentages unless indicated otherwise;

* values are median (i.q.r.). RFA, radiofrequency ablation.

† χ^2^ test, except

‡ Student's *t* test and

§ Mann–Whitney *U* test.

### Multiple imputation and balancing of co‐variables using IPTW

Data were not available for all patients; 6·1 per cent of data were missing for complications following resection of the primary colorectal tumour, 6·9 per cent for Dukes' class, 0·3 per cent for preoperative chemotherapy, 3·9 per cent for blood loss, 1·1 per cent for blood transfusion, 9·5 per cent for Pringle manoeuvre, 9·2 per cent for duration of surgery, and 0·8 per cent for length of hospital stay. These variables were imputed for subsequent analysis using the method described above.

After application of a stabilized IPTW to the data set, the co‐variables of interest were all well balanced with a standard difference of less than 0·1 (*Table* S2, supporting information)^28^. The mean(s.d.) stabilized IPTW was 0·989(0·410), indicating that the positivity assumption for IPTW had been maintained. For largest tumour less than 30 mm and largest tumour 30 mm or more subgroups, mean stabilized IPTW values were 1·003(0·250) and 1·003 (0·289) respectively.

### Correlation between type of resection, clinicopathological characteristics and surgical outcomes

Clinicopathological characteristics and surgical outcomes were compared by resection type (*Table* 1). There were no differences between the two groups except for more frequent use of the Pringle manoeuvre during NAR (*P* = 0·010) and larger tumours in the AR group (*P* < 0·001). There was also a significant association between resection type and year of liver surgery (*P* = 0·005), with a shift to predominantly NAR in later years (*Fig*. 1).

The two groups did not differ significantly in margin status, postoperative bleeding or bile leak rates, but did differ significantly on all other reported outcomes, favouring NAR. The overall morbidity rate was lower in the NAR group than in the AR group (31·1 *versus* 44·4 per cent respectively; *P* = 0·037). There were fewer major complications in the NAR group (10·4 *versus* 20·6 per cent; *P* = 0·006). Five patients (all in the AR group) experienced postoperative liver failure during the study interval.

### Overall and disease‐free survival

After excluding five perioperative deaths, 353 patients were included in the OS and 350 in the DFS analysis (3 patients had missing recurrence data and so DFS could not be calculated). During the study period, median OS was 65 (95 per cent c.i. 49 to 92) months, and the 5‐year OS rate was 52·3 (95 per cent c.i. 46 to 58) per cent. The median DFS was 16 (12 to 20) months, and the 5‐year DFS rate was 28·1 (23 to 33) per cent.

**Figure 2:**
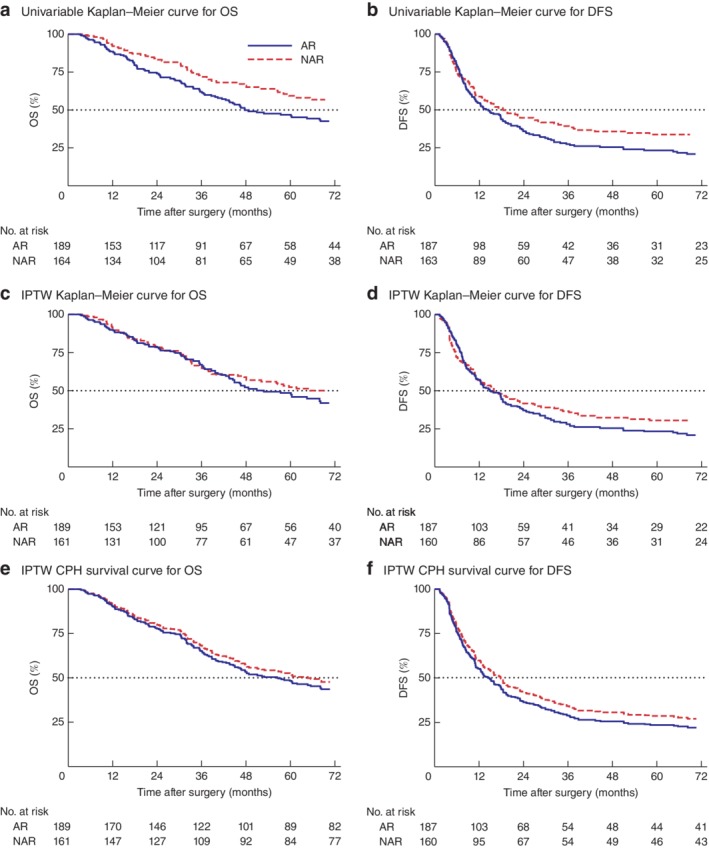
Survival curves for overall and disease‐free survival stratified by resection type. **a–d** Kaplan–Meier survival curves for overall survival (OS) and disease‐free survival (DFS) stratified by resection type in **a,b** unweighted and **c,d** inverse probability of treatment weighting (IPTW) data sets. The dotted line indicates the median value. **e,f** Survival curves for the final doubly robust Cox proportional hazards (CPH) model. AR, anatomical resection; NAR, non‐anatomical resection. **a** *P* = 0·004, **b** *P* = 0·045, **c** *P* = 0·221, **d** *P* = 0·273, **e** *P* = 0·537, **f** *P* = 0·429 (log rank test)

The unadjusted univariable analysis for OS and DFS is presented in *Table* 2. There was a significant difference in OS between AR and NAR groups (*P* = 0·004) (*Fig*. 2 *a*). Median OS was 48 months for AR and 141 months for NAR. Median DFS was longer for NAR than for AR (18 *versus* 15 months; *P* = 0·045) (*Fig*. 2 *b*).

**Table 2:** Univariable analysis of overall and disease‐free survival

		Overall survival			Disease‐free survival		
		*n*	Median(months)	*P* †	*n*	Median (months)	*P* †
Age (years)	< 55	76	101	0·155	75	18	0·941
	≥ 55 and < 65	103	80		101	18	
	≥ 65 and < 75	108	53		108	14	
	≥ 75	66	40		66	13	
Sex	F	129	106	0·059	126	22	0·049
	M	224	58		224	15	
Site of primary tumour	Colon	237	65	0·483	234	16	0·618
	Rectum	115	74		115	16	
Temporal relationship	Synchronous	180	48	0·073	177	11	0·004
	Metachronous	173	95		173	23	
Dukes' class	A/B	112	68	0·291	111	23	0·205
	C	216	60		215	15	
Primary surgery complication	No	297	68	0·082	296	18	0·088
	Yes	234	48		34	11	
Preoperative chemotherapy	No	97	68	0·624	96	23	0·111
	Yes	255	65		253	13	
Resection type	Anatomical	189	48	0·004	187	15	0·045
	Non‐anatomical	164	141		163	18	
Pringle manoeuvre	No	47	106	0·932	46	21	0·336
	Yes	272	65		270	16	
Tumour size (cm)	< 2·5	131	74	0·008	131	18	0·145
	≥ 2·5 and < 5·0	123	80		122	17	
	≥ 5·0 and < 10·0	83	39		81	10	
	≥ 10·0	16	36		16	14	
No. of tumours	Solitary	170	82	0·019	170	24	< 0·001
	Multiple	183	49		180	11	
Duration of surgery (h)*	≤ 2	93	(37)	0·013	93	19	0·157
	> 2 and ≤ 3	99	101 (30)		98	15	
	> 3 and ≤ 4	79	53 (26)		77	13	
	> 4	50	46 (18)		50	13	
Estimated blood loss (ml)	< 100	89	101	0·078	89	18	0·126
	≥ 100 and < 200	88	106		87	23	
	≥ 200 and < 400	81	60		80	15	
	≥ 400	81	46		80	10	
Blood transfusion	No	328	72	0·004	325	16	0·285
	Yes	21	31		21	16	
Hospital stay (days)	≤ 7	176	92	0·014	173	21	0·030
	> 7 and ≤ 14	146	52		146	13	
	> 14	28	46		28	9	
Complications	None or minor	296	79	0·007	293	18	0·056
	Major	57	38		57	11	
Resection margin	R0	295	80	0·011	293	18	0·027
	R1	58	43		57	11	

* Values in parentheses represent the 25 per cent quantile as the median was not reached during the study period for all groups.

† Log rank test.

### Multivariable analysis

IPTW‐adjusted Kaplan–Meier curves for OS and DFS are shown in *Fig*. 2 *c,d*. Median OS was 52 and 91 months, and median DFS was 14 and 16 months, for AR and NAR groups respectively. There was no longer a significant difference between AR and NAR for either curve (*P* = 0·221 and *P* = 0·273 respectively). The final doubly robust IPTW CPH models for OS and DFS are shown in *Table* 3. In this model, margin status was maintained based on previous research, indicating this to be a significant prognostic factor^29^, ^30^.

**Table 3:** Final doubly robust multivariable model for overall and disease‐free survival

	Hazard ratio	s.e.	*P*
Overall survival			
Resection type			0·537
Non‐anatomical	1·00 (reference)		
Anatomical	1·21 (0·62, 1·28)	0·17	
Tumour size (cm)			0·035
< 2·5	1·00 (reference)		
≥ 2·5 and < 5·0	1·09 (0·75, 1·59)	0·21	
≥ 5·0 and < 10·0	1·81 (1·13, 2·90)	0·44	
≥ 10	2·17 (1·08, 4·34)	0·77	
No. of tumours			0·035
Solitary	1·00 (reference)		
Multiple	1·48 (1·03, 2·12)	0·27	
Resection margin			0·102
R0	1·00 (reference)		
R1	1·42 (0·93, 2·16)	0·30	
Disease‐free survival			
Resection type			0·429
Non‐anatomical	1·00 (reference)		
Anatomical	1·12 (0·67, 1·18)	0·13	
Temporal relationship			0·045
Metachronous	1·00 (reference)		
Synchronous	1·33 (1·01, 1·77)	0·19	
Primary surgery complication			0·045
No	1·00 (reference)		
Yes	1·51 (1·01, 2·26)	0·31	
Complications at liver resection			0·026
None or minor	1·00 (reference)		
Major	1·49 (1·05, 2·11)	0·26	
No. of tumours			0·004
Solitary	1·00 (reference)		
Multiple	1·51 (1·14, 2·00)	0·22	
Resection margin			0·092
R0	1·00 (reference)		
R1	1·34 (0·95, 1·90)	0·24	

Values in parentheses are 95 per cent confidence intervals.

Large or multiple metastases were associated with poor OS. Synchronous or multiple liver metastases, as well as complications after resection of the primary colorectal tumour or major complications at the time of CRLM resection, persisted as significant factors associated with poor DFS. Importantly, after adjusting for confounders, neither resection type (anatomical or non‐anatomical) or margin status significantly predicted OS or DFS. *Fig*. 2 *e,f* shows the survival curves for the averaged estimates across each of the imputed data sets derived from the doubly robust IPTW CPH model.

### Recurrence pattern and salvage resection

Overall, 157 patients (43·9 per cent) developed intrahepatic recurrence, but there was no association between resection type and pattern of recurrence (intrahepatic only, extrahepatic only, or mixed) (*P* = 0·246) (*Table* 1). Of patients with intrahepatic recurrence only, 41 underwent salvage resection. This was more likely if they had NAR as initial resection rather than AR (OR 2·31, 95 per cent c.i. 1·05 to 5·11; *P* = 0·022). Although the number of patients was small, in those who did proceed to salvage liver resection there was no difference in median OS whether they had AR or NAR initially (53 *versus* 55 months respectively; *P* = 0·430) (*Fig*. S1, supporting information).

### Subgroup analysis based on size of largest tumour

There was a significant effect modification on OS (*P* = 0·011) and DFS (*P* = 0·016) for NAR based on subgrouping according to the size of the largest CRLM (*Table* S3, supporting information). Some 36·5 per cent (69 of 189) in the AR group and 61·0 per cent (100 of 164) in the NAR group had CRLMs smaller than 30 mm. Significantly more NAR procedures involving tumours of 30 mm or larger were performed over time (*P* = 0·032), reflecting the trend shown in *Fig*.  1. There was no difference in margin status between AR and NAR in either subgroup.

Patients with CRLMs smaller than 30 mm resected by a non‐anatomical approach had significantly better median OS (150 months; *P* < 0·001) and DFS (27 months; *P* = 0·016) than those with lesions smaller than 30 mm resected anatomically or with lesions of 30 mm or greater resected by either technique (*Fig*. S2a, supporting information). Similar results were observed in the IPTW‐adjusted survival curves (*Fig*. S2b, supporting information).

Multivariable analysis of the final doubly robust IPTW CPH subgroup models is shown in *Table* 4. In the largest tumour smaller than 30 mm subgroup, prognostic factors for poor OS included having an AR and multiple tumours. For DFS, having multiple tumours was the only hazardous prognostic factor. In the largest tumour at least 30 mm subgroup, complications after primary colorectal surgery and major complications following liver resection were significant for poorer OS. R1 margin status did not significantly influence survival. Major complications after liver resection as well as multiple tumours were also significantly associated with poorer DFS. For intrahepatic recurrences in the smaller than 30 mm subgroup, 27 patients underwent salvage resection. After initial NAR they were more likely to undergo repeated resection (OR 3·17, 95 per cent c.i. 1·18 to 8·47; *P* = 0·031). In the 30 mm or larger subgroup, only 14 patients underwent salvage resection and no association was found with AR or NAR at initial operation. These patients had significantly more extrahepatic recurrence than those in the smaller than 30 mm group (OR 2·06, 1·18 to 3·61; *P* = 0·011).

**Table 4:** Final doubly robust multivariable model for overall and disease‐free survival for largest tumour size subgroups

	Size of largest tumour < 30 mm			Size of largest tumour ≥ 30 mm		
	Hazard ratio	s.e.	*P*	Hazard ratio	s.e.	*P*
Overall survival						
Resection type			0·028			0·469
Non‐anatomical	1·00 (reference)			1·00 (reference)		
Anatomical	1·81 (0·33, 0·94)	0·15		0·85 (0·76, 1·81)	0·26	
Primary surgery complication						0·009
No	–			1·00 (reference)		
Yes	–			2·02 (0·29, 0·84)	0·13	
Complications at liver resection						0·006
None or minor	–			1·00 (reference)		
Major	–			1·92 (1·20, 3·08)	0·46	
No. of tumours			0·020			*‐*
Solitary	1·00 (reference)			–		
Multiple	2·02 (1·12, 3·65)	0·61		–		
Resection margin			0·968			0·070
R0	1·00 (reference)			1·00 (reference)		
R1	0·98 (0·42, 2·31)	0·43		1·53 (0·97, 2·42)	0·36	
Disease‐free survival						
Resection type			0·175			0·856
Non‐anatomical	1·00 (reference)			1·00 (reference)		
Anatomical	1·32 (0·51, 1·13)	0·15		0·97 (0·71, 1·51)	0·20	
Primary surgery complication						0·050
No	–			1·00 (reference)		
Yes	–			1·68 (0·35, 1·00)	0·16	
Complications at liver resection						0·017
None or minor	–			1·00 (reference)		
Major	–			1·65 (1·09, 2·49)	0·35	
No. of tumours			0·004			0·002
Solitary	1·00 (reference)			1·00 (reference)		
Multiple	1·87 (1·22, 2·85)	0·40		1·74 (1·22, 2·49)	0·32	
Resection margin			0·100			0·992
R0	1·00 (reference)			1·00 (reference)		
R1	1·76 (0·90, 3·44)	0·60		1·00 (0·66, 1·52)	0·21	

Values in parentheses are 95 per cent confidence intervals.

## Discussion

This study demonstrates that NAR has at least equivalent oncological outcomes to AR while proving to be safer. NAR is associated with a higher rate of salvage resection in the event of intrahepatic recurrence. Surgical complications after either primary tumour or liver resection can impact on long‐term DFS and OS. In the authors' opinion, if NAR is possible, it is the preferred approach for treating CRLM, particularly for lesions smaller than 30 mm.

The larger median size of tumours in the AR compared with the NAR group in the present study reflects the greater likelihood of these tumours abutting critical vascular or biliary structures, thereby necessitating a more extensive operation^31^. However, after adjusting for the effect of tumour size and number, the type of liver resection (AR or NAR) did not impact on either OS or DFS. There was a significant increase in the proportion of NARs undertaken over the 17‐year study interval, as well as an increase in the median size of the tumours resected non‐anatomically. This was despite no change in complexity score amongst ARs. This counters any suggestion that the NAR group is comprised only of technically easy ‘chip out’ resections. The PS IPTW analysis also controlled for any effect on outcome due to the proportional increase in NAR over time.

In subgroup analysis, AR was a poor prognostic factor for OS, but not for DFS, in patients with tumours smaller than 30 mm. This may be because patients who initially presented with small tumours were more likely to proceed to salvage resection for intrahepatic recurrence if the original resection was non‐anatomical rather than an AR, which would leave a smaller liver remnant. In the event of intrahepatic recurrence there was equivalent OS regardless of the initial approach to resection, although the number of events for analysis was small. In contrast for large (at least 30 mm) tumours, no association between salvage resection and initial resection type was observed, possibly because these patients were more likely to present with additional extrahepatic recurrence.

NAR was consistently safer than AR across all studied outcome measures including overall morbidity, mortality, blood loss, transfusion requirement and length of hospital stay. Importantly, there was no difference in bile leak rates between the two resection types. Major complications after liver resection or at the time of the primary colorectal resection influenced survival after liver resection for CRLM. This is consistent with findings from a study^32^ using pooled individual patient data from three large phase III clinical trials of patients who had curative resection of a primary colorectal cancer. The relationship between surgical complications and oncological outcome is not fully understood, but is likely to involve numerous immune regulatory mechanisms and release of inflammatory and growth factors^33^, ^34^.

The original hypothesis of the present study was not supported by the finding that a microscopically positive resection (R1) margin did not predict DFS or OS in any of the multivariable models. This remains a contentious and unresolved issue in the surgical literature. Although several large studies^29^, ^35^, ^36^, ^37^ have shown that an R1 resection does increase the risk of tumour recurrence, others^38^, ^39^ did not observe this. Regardless, the predominant paradigm is that evidence of residual disease at the resection margin increases the risk of tumour recurrence. Patients with large tumours had a higher rate of adjacent micrometastases compared with those with small tumours^40^, ^41^. Tumour biology may eclipse the impact of relatively few residual tumour cells at the surgical margin^29^. In the present model, it is likely that margin status was outperformed by tumour size and number, possibly as a surrogate of tumour biology, in determining long‐term outcome. Multiple or large metastases were poor prognostic factors for OS, whereas multiple metastases or synchronous bowel and liver resection, as well as complications after primary resection and major complications after liver resection, were poor prognostic factors for DFS.

Limitations of this study include its retrospective design and lack of genetic data in the analysis. A recent study^42^ showed that amongst *KRAS* mutant tumours, which are known to be associated with increased rates of vascular invasion, AR conferred a benefit to intrahepatic recurrence and overall DFS. Interestingly, even in the *KRAS* mutant subgroup, R1 status did not predict DFS. Although *KRAS* mutations are present in 30–40 per cent of colorectal tumours with liver metastases, there are many other potential mutational combinations in CRLMs. Future studies will need to consider stratifying patients according to mutation status, as well as other indicators of underlying tumour biology.

## Supporting information


**Table S1** Types of anatomical liver resection
**Table S2** Standardized differences in baseline co‐variables used in multivariable logistic regression model of propensity score, before and after inverse probability of treatment weighting, for the whole cohort and for each subgroup based on maximum tumour diameter
**Table S3** Summary of univariable analyses of overall and disease‐free survival for largest tumour size <30 mm and ≥ 30 mm subgroups performed by log rank test
**Fig. S1** Univariable overall survival (OS) curves comparing anatomical and non‐anatomical hepatic resection after salvage resection for intrahepatic recurrence
**Fig. S2** Kaplan–Meier survival curves for overall survival (OS) and disease‐free survival (DFS) stratified by resection type and subgroup based on the diameter of the largest resected tumour in both the unweighted (A) and IPTW (B) data sets
