# Supplementary material for: Propensity score analysis of non‐anatomical versus anatomical resection of colorectal liver metastases
Source: BJS Open. 2019 Mar 18;3(4):521–31. doi: 10.1002/bjs5.50154 (PMC6677098; doi:10.1002/bjs5.50154)
Supplement: Supplementary file 1 — Table S1 Types of anatomical liver resection Table S2 Standardized differences in baseline co‐variables used in multivariable logistic regression model of propensity score, before and after inverse probability of treatment weighting, for the whole cohort and for each subgroup based on maximum tumour diameter Table S3 Summary of univariable analyses of overall and disease‐free survival for largest tumour size <30 mm and ≥ 30 mm subgroups performed by log rank test Fig. S1 Univariable overall survival (OS) curves comparing anatomical and non‐anatomical hepatic resection after salvage resection for intrahepatic recurrence Fig. S2 Kaplan–Meier survival curves for overall survival (OS) and disease‐free survival (DFS) stratified by resection type and subgroup based on the diameter of the largest resected tumour in both the unweighted (A) and IPTW (B) data sets [file BJS5-3-521-s001.docx]

**BJS5_50154**

**Propensity score analysis of non-anatomical *versus* anatomical resection of colorectal liver metastases**

**K. M. Brown, M. F. Albania, J. S. Samra, P. J. Kelly and T. J. Hugh**

| **Table S1 Types of anatomical liver resection** | | |
| --- | --- | --- |
| **Resection type** | **No. of resections** | |
|  | **(n=194)** | **(%)** |
| Extended left hepatectomy | 10 | 5.2 |
| Extended right hepatectomy | 30 | 15.5 |
| Left hepatectomy | 27 | 13.9 |
| Right hepatectomy | 75 | 38.7 |
| Central liver resection | 5 | 2.6 |
| Right posterior sectionectomy | 7 | 3.6 |
| Right anterior sectionectomy | 1 | 0.5 |
| Left lateral sectionectomy | 37 | 19.1 |
| Caudate resection | 2 | 1 |

| **Table S2 Standardized differences in baseline co-variables used in multivariable logistic regression model of propensity score, before and after inverse probability of treatment weighting, for the whole cohort and for each subgroup based on maximum tumour diameter** | | | | | | | | |
| --- | --- | --- | --- | --- | --- | --- | --- | --- |
|  |  |  |  |  |  |  |  |  |
|  |  |  |  |  |  |  |  |  |
| **Covariate** |  | **Unweighted** | | |  | **IPTW** | | |
|  |  |  |  |  |  |  |  |  |
|  |  | **Mean in treated** | **Mean in untreated** | **Standardised difference** |  | **Mean in treated** | **Mean in untreated** | **Standardised difference** |
| ***Standardised differences for the whole cohort*** | | | |  |  |  |  |  |
| Age category |  | 2.47 | 2.51 | -0.047 |  | 2.45 | 2.47 | -0.028 |
| Sex |  | 1.38 | 1.36 | 0.039 |  | 1.37 | 1.36 | 0.017 |
| Synchronous |  | 1.47 | 1.5 | -0.059 |  | 1.48 | 1.49 | -0.029 |
| Year of liver operation category | | 2.29 | 2.13 | 0.244 |  | 2.16 | 2.22 | -0.088 |
| Pre-operative chemotherapy |  | 1.28 | 1.27 | 0.017 |  | 1.27 | 1.27 | -0.005 |
| Number of tumours |  | 1.49 | 1.51 | -0.043 |  | 1.51 | 1.51 | -0.005 |
| Tumour size |  | 1.67 | 2.21 | -0.642 |  | 1.9 | 1.95 | -0.059 |
| Pringle |  | 1.09 | 1.2 | -0.292 |  | 1.14 | 1.15 | -0.043 |
|  |  |  |  |  |  |  |  |  |
| ***Standardised differences for patients with maximum tumour diameter <30mm*** | | | | | | | |  |
| Age category |  | 2.42 | 2.07 | 0.375 |  | 2.3 | 2.33 | -0.032 |
| Sex |  | 1.34 | 1.38 | -0.069 |  | 1.35 | 1.36 | -0.022 |
| Synchronous |  | 1.47 | 1.42 | 0.109 |  | 1.45 | 1.46 | -0.028 |
| Year of liver operation category | | 2.31 | 2.17 | 0.205 |  | 2.26 | 2.28 | -0.029 |
| Pre-operative chemotherapy |  | 1.21 | 1.16 | 0.135 |  | 1.2 | 1.22 | -0.042 |
| Number of tumours |  | 1.53 | 1.57 | -0.08 |  | 1.54 | 1.54 | 0.002 |
|  |  |  |  |  |  |  |  |  |
| ***Standardised differences for patients with maximum tumour diameter ≥30mm*** | | | | | | | |  |
| Age category |  | 2.5 | 2.71 | -0.192 |  | 2.66 | 2.64 | 0.022 |
| Sex |  | 1.42 | 1.34 | 0.165 |  | 1.34 | 1.36 | -0.045 |
| Synchronous |  | 1.47 | 1.55 | -0.162 |  | 1.55 | 1.53 | 0.045 |
| Year of liver operation category | | 2.27 | 2.01 | 0.383 |  | 2.11 | 2.11 | 0.006 |
| Pre-operative chemotherapy |  | 1.39 | 1.33 | 0.119 |  | 1.33 | 1.35 | -0.044 |
| Number of tumours |  | 1.48 | 1.5 | -0.031 |  | 1.53 | 1.5 | 0.05 |

| **Table S3 Summary of univariable analyses of overall and disease-free survival for largest tumour size < 30 mm and ≥ 30 mm subgroups performed by log rank test** | | | | | | | | | | | | | | |
| --- | --- | --- | --- | --- | --- | --- | --- | --- | --- | --- | --- | --- | --- | --- |
| **(5 perioperative deaths excluded)** | | | | | | | | |  | |  |  |  | |
| **Variable** | ***Overall Survival*** | | | | | | ***Disease Free Survival*** | | | | | | | |
|  | **Size of Largest Tumour <30mm** | | | **Size of Largest Tumour ≥30mm** | | | **Size of Largest Tumour <30mm** | | | | **Size of Largest Tumour ≥30mm** | | | |
|  | **n=169**  **(48%)** | **Median OS (months)** | **P value** | **n=184**  **(52%)** | **Median OS (months)** | **P value** | **n=169**  **(48%)** | **Median OS (months)** | | **P value** | **n=184**  **(52%)** | **Median OS (months)** | | **P value** |
| ***Clinicopathological*** |  |  |  |  |  |  |  |  | |  |  |  | |  |
| **Age (years)** |  |  | 0.38 |  |  | 0.86 |  |  | | 0.88 |  |  | | 0.72 |
| <55 | 40 | (38) |  | 36 | 41 |  | 40 | 26 | |  | 35 | 10 | |  |
| ≥55 & <65 | 60 | 91 |  | 34 | 79 |  | 59 | 18 | |  | 42 | 14 | |  |
| ≥65 & <75 | 51 | 60 |  | 57 | 47 |  | 51 | 15 | |  | 57 | 14 | |  |
| ≥75 | 18 | 56 |  | 48 | 37 |  | 18 | 19 | |  | 48 | 11 | |  |
| **Sex** |  |  | 0.19 |  |  | 0.14 |  |  | | 0.063 |  |  | | 0.34 |
| Female | 61 | 160 |  | 68 | 82 |  | 60 | 32 | |  | 66 | 12 | |  |
| Male | 108 | 67 |  | 116 | 37 |  | 108 | 16 | |  | 116 | 14 | |  |
| **Site of primary tumour** |  |  | 0.26 |  |  | 0.97 |  |  | | 0.95 |  |  | | 0.52 |
| Colon | 114 | 95 |  | 123 | 47 |  | 113 | 18 | |  | 121 | 13 | |  |
| Rectum | 54 | 91 |  | 61 | 37 |  | 52 | 22 | |  | 61 | 14 | |  |
| **Temporal relationship** |  |  | 0.060 |  |  | 0.26 |  |  | | **0.024** |  |  | | **0.028** |
| Synchronous | 92 | 60 |  | 88 | 33 |  | 91 | 13 | |  | 86 | 9 | |  |
| Metachronous | 77 | 160 |  | 96 | 52 |  | 77 | 33 | |  | 96 | 20 | |  |
| **Dukes stage** |  |  | 0.40 |  |  | 0.31 |  |  | | 0.46 |  |  | | 0.12 |
| A/B | 45 | 160 |  | 67 | 52 |  | 45 | 33 | |  | 66 | 19 | |  |
| C | 110 | 95 |  | 106 | 35 |  | 109 | 18 | |  | 106 | 10 | |  |
| **Primary surgery complication** |  |  | 0.41 |  |  | **0.039** |  |  | | 0.89 |  |  | | 0.061 |
| No | 147 | 95 |  | 150 | 46 |  | 11 | 19 | |  | 150 | 15 | |  |
| Yes | 11 | 160 |  | 23 | 32 |  | 146 | 27 | |  | 23 | 8 | |  |
| **Pre-operative chemotherapy** |  |  | 0.72 |  |  | 0.13 |  |  | | 0.21 |  |  | | **0.046** |
| No | 32 | 160 |  | 65 | 68 |  | 31 | 33 | |  | 65 | 19 | |  |
| Yes | 136 | 95 |  | 119 | 35 |  | 136 | 17 | |  | 117 | 10 | |  |
| **Resection type** |  |  | **0.020** |  |  | 0.38 |  |  | | 0.18 |  |  | | 0.57 |
| Anatomical | 69 | 60 |  | 120 | 42 |  | 69 | 17 | |  | 118 | 12 | |  |
| Non-anatomical | 100 | 160 |  | 64 | 52 |  | 99 | 27 | |  | 64 | 14 | |  |
| **Pringle used** |  |  | 0.31 |  |  | 0.80 |  |  | | 0.12 |  |  | | 0.97 |
| No | 20 | (74) |  | 27 | 48 |  | 20 | 43 | |  | 26 | 12 | |  |
| Yes | 132 | 95 |  | 140 | 43 |  | 131 | 17 | |  | 139 | 14 | |  |
| **Number of tumours** |  |  | **0.010** |  |  | 0.16 |  |  | | **<0.001** |  |  | | **0.001** |
| Solitary | 77 | 160 |  | 93 | 52 |  | 77 | 160 | |  | 93 | 21 | |  |
| Multiple | 92 | 61 |  | 91 | 31 |  | 91 | 13 | |  | 89 | 7 | |  |
|  |  |  |  |  |  |  |  |  | |  |  |  | |  |
| ***Surgical Outcomes*** |  |  |  |  |  |  |  |  | |  |  |  | |  |
| **Length of operation (hours)** |  |  | 0.087 |  |  | 0.33 |  |  | | 0.24 |  |  | | 0.50 |
| ≤2 | 53 | (57) |  | 40 | 59 |  | 53 | 26 | |  | 40 | 18 | |  |
| >2 & ≤3 | 44 | (40) |  | 55 | 52 |  | 44 | 22 | |  | 54 | 11 | |  |
| >3 & ≤4 | 36 | 64 |  | 43 | 42 |  | 35 | 11 | |  | 43 | 16 | |  |
| >4 | 17 | 58 |  | 33 | 33 |  | 17 | 17 | |  | 33 | 8 | |  |
| **Estimated blood loss (ml)** |  |  | 0.89 |  |  | 0.33 |  |  | | 0.57 |  |  | | 0.47 |
| <100 | 54 | (47) |  | 35 | 59 |  | 54 | 18 | |  | 35 | 21 | |  |
| ≥100 & <200 | 46 | 160 |  | 42 | 82 |  | 46 | 36 | |  | 41 | 16 | |  |
| ≥200 & <400 | 35 | 91 |  | 46 | 37 |  | 35 | 13 | |  | 45 | 14 | |  |
| ≥400 | 26 | 95 |  | 55 | 36 |  | 25 | 14 | |  | 55 | 9 | |  |
| **Blood transfusion** |  |  | 0.77 |  |  | 0.013 |  |  | | 0.82 |  |  | | 0.32 |
| No | 162 | 95 |  | 166 | 48 |  | 161 | 18 | |  | 164 | 12 | |  |
| Yes | 4 | 95 |  | 17 | 24 |  | 4 | 26 | |  | 17 | 14 | |  |
| **Hospital stay (days)** |  |  | 0.16 |  |  | **0.009** |  |  | | 0.15 |  |  | | 0.086 |
| ≤7 | 94 | 160 |  | 82 | 52 |  | 93 | 27 | |  | 80 | 16 | |  |
| >7 & ≤14 | 63 | 64 |  | 83 | 38 |  | 62 | 12 | |  | 83 | 12 | |  |
| >14 | 10 | (45) |  | 18 | 23 |  | 10 | 10 | |  | 18 | 7 | |  |
| **Complications** |  |  | 0.79 |  |  | **0.008** |  |  | | 0.89 |  |  | | 0.053 |
| None/Minor | 151 | 95 |  | 145 | 52 |  | 150 | 19 | |  | 143 | 16 | |  |
| Major | 18 | (35) |  | 39 | 31 |  | 18 | 15 | |  | 39 | 8 | |  |
| **Resection margin** |  |  | 0.51 |  |  | 0.087 |  |  | |  |  |  | |  |
| R0 | 151 | 160 |  | 144 | 48 |  | 150 | 22 | | **0.012** | 143 | 14 | | 0.67 |
| R1 | 18 | 95 |  | 40 | 33 |  | 18 | 9 | |  | 39 | 10 | |  |
| Values in parentheses represent 25% quantile as the median was not reached within the study period for all groups | | | | | | | | | | | | | | |

**Fig. S1 Univariable overall survival (OS) curves comparing anatomical and non-anatomical hepatic resection after salvage resection for intrahepatic recurrence**


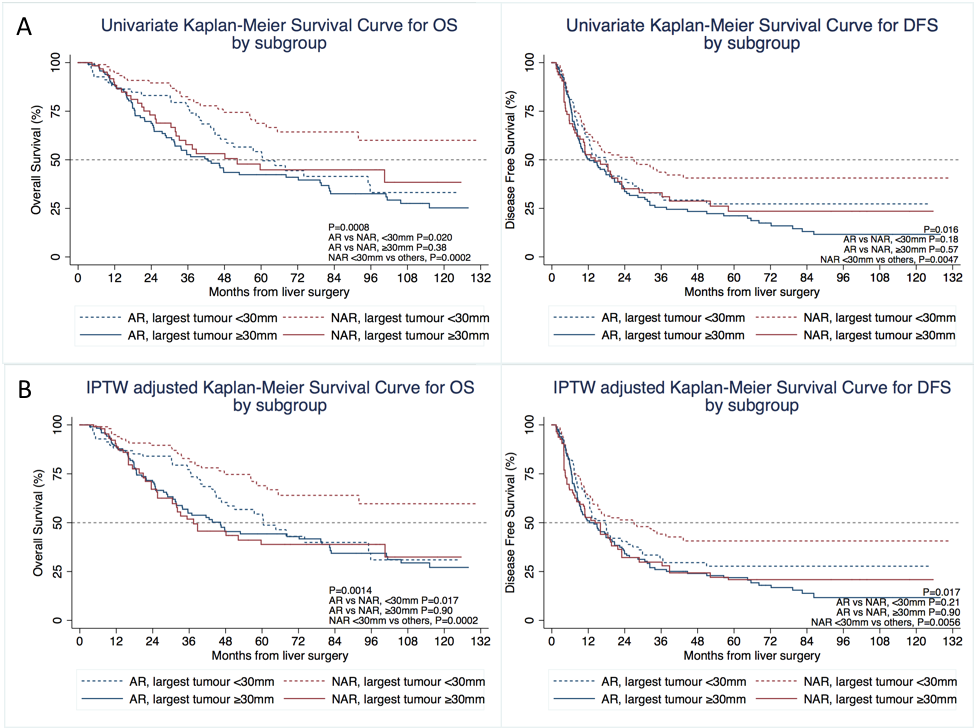


**Fig. S2 Kaplan–Meier survival curves for overall survival (OS) and disease-free survival (DFS) stratified by resection type and subgroup based on the diameter of the largest resected tumour in both the unweighted (A) and IPTW (B) data sets**
